# NIA Division of Geriatrics and Clinical Gerontology

**DOI:** 10.1093/geroni/igaf122.1557

**Published:** 2025-12-31

**Authors:** Chhanda Dutta

**Affiliations:** National Institute on Aging, Rockville, Maryland, United States

## Abstract

Dr. Dutta will discuss research foci of the NIA Division of Geriatrics and Clinical Gerontology. Dr. Dutta will also be available for small group discussion.

